# Correspondence: Statistical errors in “Factors influencing fear of cancer recurrence among prostate cancer patients: a systematic review and meta analysis” by Ziyi Qi, Sijia Hou, Ying Zhang, Siyuan Wu, and Wei Wang

**DOI:** 10.1007/s00520-025-09977-7

**Published:** 2025-10-10

**Authors:** Gregor Weißflog

**Affiliations:** Medical Psychology and Medical Sociology, University of Leipzig, Leipzig, Germany

Dear Chief Editor Prof. Ashbury and authors,

I have read with great interest the article “Factors influencing fear of cancer recurrence among prostate cancer patients: a systematic review and meta analysis” by Ziyi Qi, Sijia Hou, Ying Zhang, Siyuan Wu & Wei Wang published in Supportive Care in Cancer (Web: 10.1007/s00520-025–09465-y). The article addresses an important topic. However, I would like to point out that I noticed several statistical errors and methodological inconsistencies in the article that could compromise the validity of the results.

Within the original article, the results of the meta-analysis are presented in Table 3 (page 7 of 12). Odds ratios (OR) and their 95% confidence intervals (95% CI) for predictors of fear of cancer recurrence are presented. Predictors for which the 95% CI contains ‘1’ are presented as significant. In my statistical understanding, this is wrong.

The following self-created table lists the predictors for which I consider the statistical evaluation to be incorrect.

**Table:** 

*Influence factor*	*OR [95% CI]*	*p*	*Quote from text*
*Age*	*0.9084* *[0.7380; 1.0781]*	< *0.0001*	*„showed that age was confirmed to be a factor affecting FCR “*
*Social support*	*1.2149* *[0.8543; 1.5755]*	< *0.0001*	*„indicating that higher levels of support [and hope] were associated with lower FCR scores “*
*Marriage/partnership*	*1.0462* *[0.9626; 1.1297]*	< *0.0001*	*„four studies indicated that men with prostatae cancer who had a partner experienced lower FCR “*
*Income*	*0.9076* *[0.7765; 1.0386]*	< *0.0001*	*„Furthermore, income demonstrated a negative association with FCR in prostate cancer patients, while employment status showed a positive association.“*
*Employment status*	*1.0799* *[0.9800; 1.1798]*	< *0.0001*	
*BCR*	*3.2214* *[0.4982; 5.9446]*	*0.0204*	*„Disease factors: Prostate cancer patients with a longer time since diagnosis,…, experience of BCR, …, and poor urinary and hormone function tended to have higher FCR*
Treatment therapy	2.0567 [0.4361; 3.6774]	0.0129	
Hormone function	0.8042 [0.4259; 1.1825]	< 0.0001	
Urinary function	1.1271 [0.8577; 1.3965]	< 0.0001	
Time since diagnosis	1.5252 [0.1540; 2.8964]	0.0293	

As a result, this incorrect significance interpretation of the predictors forms the basis for the interpretation of the factors that influence fear of cancer recurrence in prostate cancer patients. Against this background, the significance of the entire article would have to be assessed as very limited and questionable.

The methods section contains only sparse information on the associated methodological procedure, which does not allow any conclusions to be drawn about a different significance assessment of the ORs compared to the usual assessment as described above. Since the systematic review/meta-analysis was registered with Prospero (Web: https://www.crd.york.ac.uk/PROSPERO/view/CRD42024497270), further information on the methodological approach was sought there. However, no further information was found. My question here is: In this context, what explanation allows the OR to be classified as significant if the 95% confidence interval includes the number 1?

Further, the authors quote within the method section “All reported effect sizes were transformed into ORs.” My question here is: Which effect sizes were calculated for which predictors (taking into account the scale level, nominal, ordinal, metric) and how were these effect sizes transformed into ORs?

Finally, I would recommend reviewing the statistical analysis and addressing the issues mentioned in order to strengthen the validity of the results. I would appreciate hearing the authors' comments on this matter.

Best regards,

Gregor Weißflog.

PhD, University of Leipzig, Germany

## Data Availability

No datasets were generated or analysed during the current study.

